# Enhanced photocatalytic water splitting of a SILAR deposited α-Fe_2_O_3_ film on TiO_2_ nanoparticles

**DOI:** 10.1039/c9ra05155d

**Published:** 2019-10-07

**Authors:** Zahra-Sadat Pourbakhsh, Kyana Mohammadi, Ahmad Moshaii, Maryam Azimzadehirani, Amir Hosseinmardi

**Affiliations:** Department of Physics, Tarbiat Modares University P.O Box 14115-175 Tehran Iran moshaii@modares.ac.ir; Department of Chemistry, Tarbiat Modares University P.O Box 14115-175 Tehran Iran

## Abstract

We have investigated the effect of deposition of a α-Fe_2_O_3_ thin layer on a substrate of TiO_2_ nanoparticles for photoelectrochemical (PEC) water splitting. The TiO_2_ layer was coated on an FTO substrate using the paste of TiO_2_ nanoparticles. The α-Fe_2_O_3_ layer was deposited on the TiO_2_ thin film, using the method of Successive Ionic Layer Adsorption and Reaction (SILAR) with different cycles. Various characterizations including XRD, EDX and FE-SEM confirm the formation of α-Fe_2_O_3_ and TiO_2_ nanoparticles on the electrode. The UV-visible absorption spectrum confirms a remarkable enhancement of the absorption of the α-Fe_2_O_3_/TiO_2_/FTO composite relative to the bare TiO_2_/FTO. In addition, the photocurrents of the composite samples are remarkably higher than the bare TiO_2_/FTO. This is mainly due to the low band gap of α-Fe_2_O_3_, which extends the absorption spectrum of the α-Fe_2_O_3_/TiO_2_ composite toward the visible region. In addition, the impedance spectroscopy analysis shows that the recombination rate of the charge carriers in the α-Fe_2_O_3_/TiO_2_ is lower than that for the bare TiO_2_. The best PEC performance of the α-Fe_2_O_3_/TiO_2_ sample was achieved by the sample of 70 cycles of α-Fe_2_O_3_ deposition with about 7.5 times higher photocurrent relative to the bare TiO_2_.

## Abstract 2

Optimization of photoelectrochemical water splitting by a composite of SILAR-deposited α-Fe_2_O_3_ thin film on a substrate of TiO_2_ nanoparticles.

## Graphical abstract

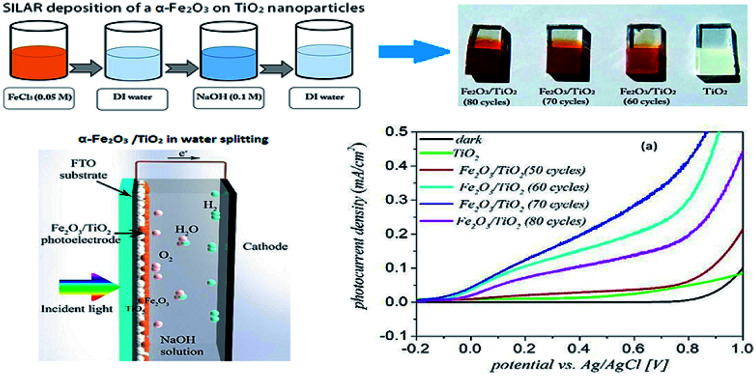

## Introduction

Fossil fuels are currently the main non-renewable energy resource in the world, and have negative effects on the environment like greenhouse gas production and global temperature rise. These unclean fuels cannot meet the continuously increasing future demands for energy resources.^1–4^ Hydrogen as a non-polluting fuel can be considered as a good candidate of sustainable energy resources.^5^ Of various methods to produce hydrogen, efficient photoelectrochemical (PEC) water splitting provides a promising pathway for solar-to-hydrogen conversion using cost-effective semiconductor materials.^6–9^

In PEC water splitting, hydrogen is generated by the absorption of light in a semiconductor, which must have a suitable bandgap and suitable energies of conduction and valance bands relative to the oxidation and reduction potentials of water. Also, it should be chemically stable in the electrolyte of the PEC process.^10–12^

In recent years, TiO_2_ nanoparticles have been extensively studied for PEC water splitting due to their low cost, simple preparation, non-toxicity and chemical stability. However, the high bandgap of TiO_2_ (3.2 eV for the anatase and 3.0 eV for the rutile phase) only allows absorption of the ultraviolet region of sun light, which covers only 5% of the solar spectrum. This significantly decreases the efficiency of PEC using the TiO_2_ nanostructures. To improve the PEC properties of TiO_2_, many methods such as sensitization to the sunlight with doping of nitrogen or heavy metals^13–17^ and incorporating plasmonic nanoparticles such as Ag, Au, Pt^18–20^ have been investigated in the literatures. Most of these approaches have the drawback of increasing crystal deformations or introducing defects that act as additional electron–hole recombination centers.^21–26^ In addition, combining TiO_2_ with another semiconductor with suitable band alignments has been reported to achieve larger electron/hole separation due to the movement of charge carriers between the different semiconductors. With this regard, Fe_2_O_3_ with a bandgap of about 1.9–2.2 eV, which can absorb photons of visible light spectrum,^27,28^ is a suitable semiconductor to be mixed with TiO_2_ to extend the adsorption spectrum of the composite efficiency.^29^ However, two main disadvantages of Fe_2_O_3_ are the low conductivity and a short diffusion length of excitons (2–20 nm), which significantly diminish its photocatalytic efficiency.^30,31^ However, these deficiencies of Fe_2_O_3_ can be resolved by combining TiO_2_ with Fe_2_O_3_, due to a strong electric field at the interface of Fe_2_O_3_/TiO_2_.^30–32^ Accordingly, the combination of these two semiconductors has attracted many attentions recently.^33–43^

**Fig. 1:**
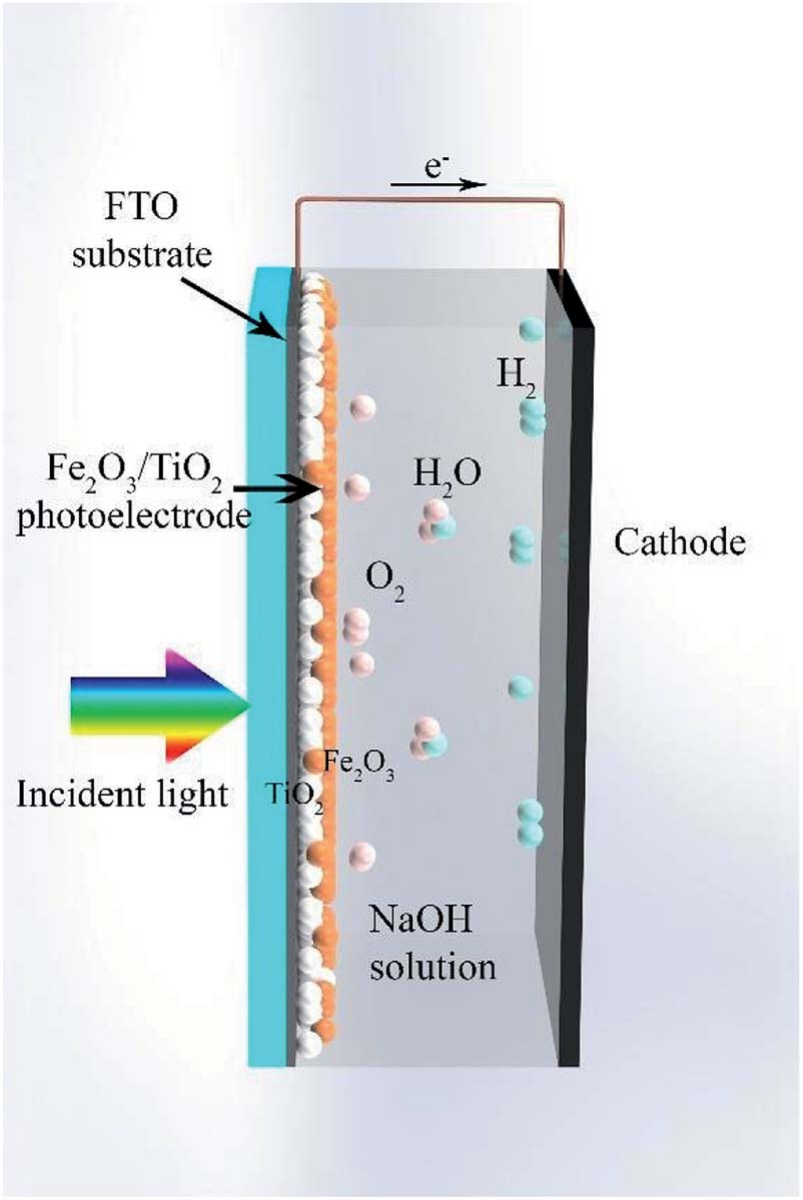
Schematic of the composite electrode of α-Fe_2_O_3_/TiO_2_ in the water splitting process.

In this work, a composite electrode of α-Fe_2_O_3_/TiO_2_ was introduced as a photoanode for PEC cells. The SILAR method was used for the deposition of α-Fe_2_O_3_ on the TiO_2_ substrate. Fig. 1 shows a schematic of the composite electrode in the water-splitting process. Under visible light irradiation, the composite electrode produces electron–hole pairs and the holes oxidize water at the electrode surface to generate oxygen. The produced electrons migrate to the counter electrode and take part in the reduction of water to produce hydrogen. We studied the optimum thickness of α-Fe_2_O_3_ on the TiO_2_ substrate to obtain the best water-splitting activity. We found that under 70-cycle deposition of α-Fe_2_O_3_, the best PEC water-splitting activity occurs, which corresponds to about 7.5 times photocurrent improvement relative to the bare TiO_2_ electrode. This result is mainly due to extension of the absorption spectrum of the sample into the visible light, due to incorporating α-Fe_2_O_3_. In addition, the recombination of charge carriers greatly decreases by using the α-Fe_2_O_3_/TiO_2_ composite.

## Experimental section

**Fig. 2:**
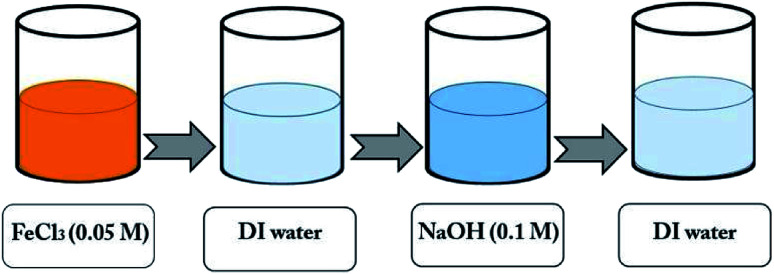
A schematic of the SILAR process for deposition of α-Fe_2_O_3_ nanoparticles on a sample for one cycle.

**Fig. 3:**
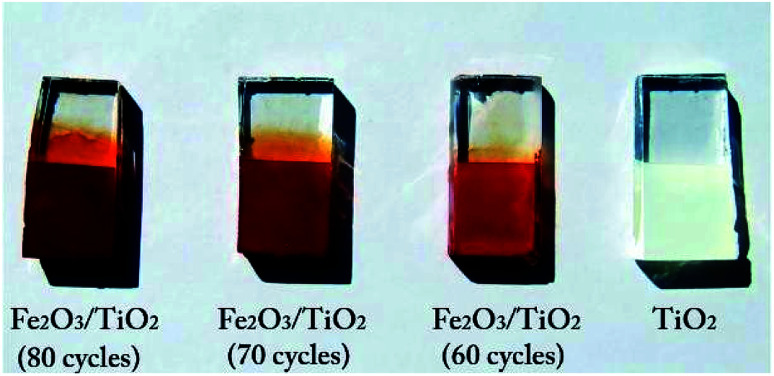
A photograph of the samples of α-Fe_2_O_3_/TiO_2_/FTO with different SILAR deposition cycles of α-Fe_2_O_3_.

The TiO_2_ layer was deposited on an area of 1 cm × 1 cm of the FTO substrate (with the sizes of 1 cm × 2 cm) by the doctor blade method,^44^ using the paste of TiO_2_ (Degussa, P-25) purchased from Sharifsolar. Then, a thin film of α-Fe_2_O_3_ nanoparticles was deposited on the TiO_2_ layer by the SILAR method, which provides a strong control over the film thickness. In this method, 0.05 M FeCl_3_ and 0.1 M NaOH, were used as iron and hydroxide precursors.^42^ The synthesis process is shown in Fig. 2, in which each immersion stage lasted 10 seconds. After depositing 30 cycles, the solutions of FeCl_3_ and NaOH were renewed by the fresh ones. The deposition of Fe_2_O_3_ on the samples by the SILAR method was done with various cycles of 50, 60, 70 and 80^th^. After finishing the SILAR deposition, we completely remove the tiny layer of α-Fe_2_O_3_ on the back of FTO by ethanol. Then the composite samples were placed inside a furnace for annealing at 550 °C for 4 hours. Fig. 3 shows a photo of the samples fabricated by the SILAR method with different cycles.

For the electrochemical measurements, the entire substrate was illuminated by a 100 W Xe lamp, so the illumination area of the α-Fe_2_O_3_/TiO_2_ layer was 1 cm × 1 cm. In addition, the illumination was frontside.

## Results and discussion

### Characterization

**Fig. 4:**
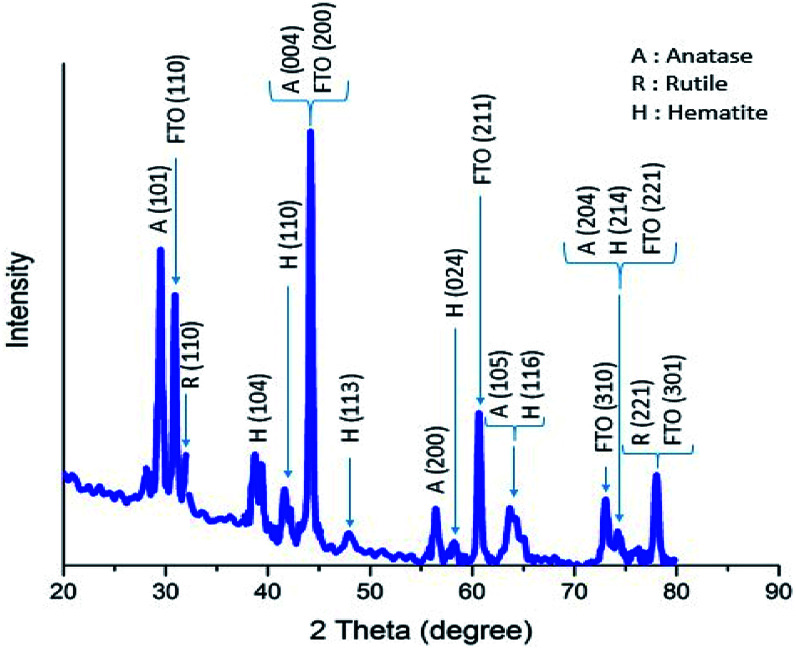
XRD diffraction pattern of a sample of α-Fe_2_O_3_/TiO_2_/FTO prepared with 70 SILAR cycles.

The X-ray diffraction pattern of a sample of α-Fe_2_O_3_/TiO_2_/FTO prepared with 70 SILAR cycles is shown in Fig. 4, using an X-ray diffractometer (Xpert, Philips) with Cu Kα radiation (*λ* = 1.54 Å). According to the reference cards of (JCPD-01-083-2243) and (JCPD-01-073-2224), the peaks of the crystalline phases of anatase and rutile of TiO_2_ are clearly seen at the 2*θ* angles of 29.5°, 44.2°, 56.4°, 63.5°, 74.4°, 31.9°, 77.8° relating to the planes of (101), (004), (200), (105), (204), (110), and (221), respectively. The substrate peaks of SnO_2_ are identified, in the pattern, according to the card of (JCPD-01-077-0450). On the other hand, the diffraction peaks of α-Fe_2_O_3_ nanoparticles are characterized at the angles of 38.8°, 41.7°, 47.9°, 58.2° and 74.1°, relating to the planes of (104), (110), (113), (024), (116) and (214), according to the card of (JCPD-01-086-0550).

**Fig. 5:**
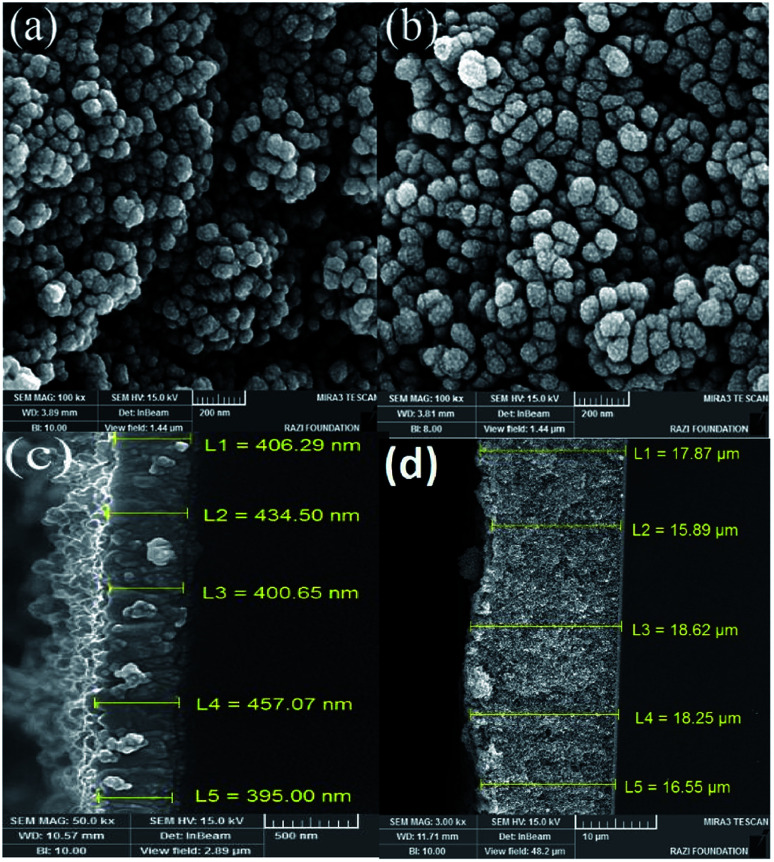
FE-SEM images from various samples of (a) TiO_2_/FTO, (b) α-Fe_2_O_3_/TiO_2_/FTO with 70 SILAR cycles, (c) cross-sectional image of TiO_2_/FTO, (d) cross-sectional image of α-Fe_2_O_3_/TiO_2_/FTO with 70 SILAR cycles.

In order to investigate the morphology of the samples, the Field Emission Scanning Electron Microscopy (FESEM) of the samples was taken using the MIRA3 TESCAN microscope. The top and the cross-section images of the TiO_2_ samples are shown in Fig. 5. We see that the thickness of the TiO_2_ layer on the surface is around 400 nm. While a very thick layer of Fe_2_O_3_ (about 18 μm) is produced on the sample by the 70-cycle SILAR deposition.

**Fig. 6:**
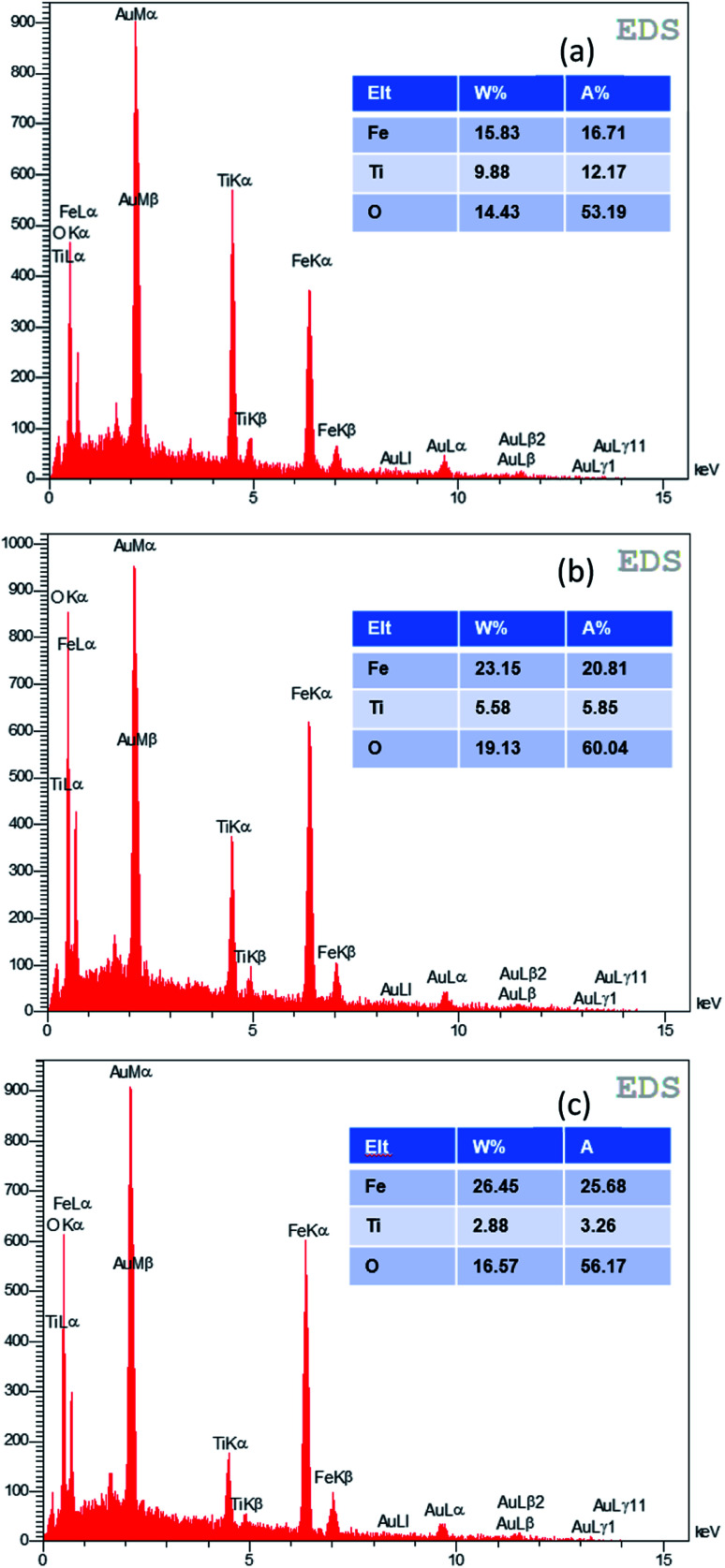
EDX analysis of various samples of α-Fe_2_O_3_/TiO_2_/FTO with (a) 60, (b) 70, (c) 80 SILAR cycles.

The EDX analysis was used for elemental determination of the samples. The weight percentages of the elements Ti, O, Fe and Au (which is used for preparation of the layers for EDX/FESEM) for the samples with different SILAR cycles are shown in Fig. 6. It is seen that the iron percentage of the best α-Fe_2_O_3_/TiO_2_ sample for the PEC application (which is the 70-cycle sample) was about 23.15% (wt).

**Fig. 7:**
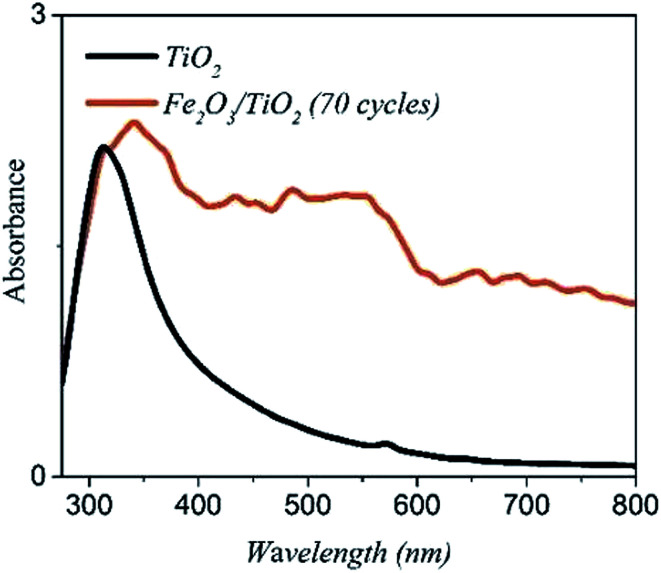
The UV-vis spectra of the samples of TiO_2_/FTO and α-Fe_2_O_3_/TiO_2_/FTO with 70-cycle.

Fig. 7 shows the UV-vis absorption spectrums of the bare TiO_2_ and the α-Fe_2_O_3_/TiO_2_ sample with the 70-cycle. The bare TiO_2_ has an absorption edge around 390 nm (∼3.13 eV), which is related to its wide bandgap. The absorption edge of α-Fe_2_O_3_/TiO_2_ has been transferred to the visible range with a much higher absorption intensity than the bare TiO_2_ in the range of 400–800 nm.

**Fig. 8:**
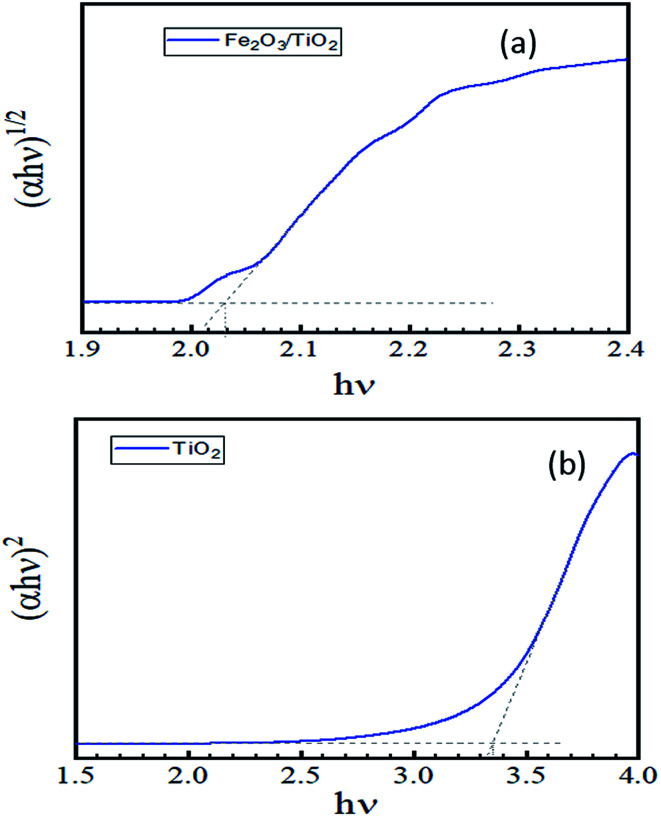
Tauc plot calculated to find the band gap of the layer from the UV-vis spectrum (a) α-Fe_2_O_3_/TiO_2_ (b) pure TiO_2_.

The bandgaps of the fabricate TiO_2_ and Fe_2_O_3_/TiO_2_ film are calculated using the Tauc plot. As it showed in Fig. 8, the tangent lines indicates that the Fe_2_O_3_ has an indirect band gap of about 2.04 eV and direct band gap of TiO_2_ is about 3.35 eV for our sample; which is agreed with other observations.^42,45^

### Photoelectrochemical characterization

**Fig. 9:**
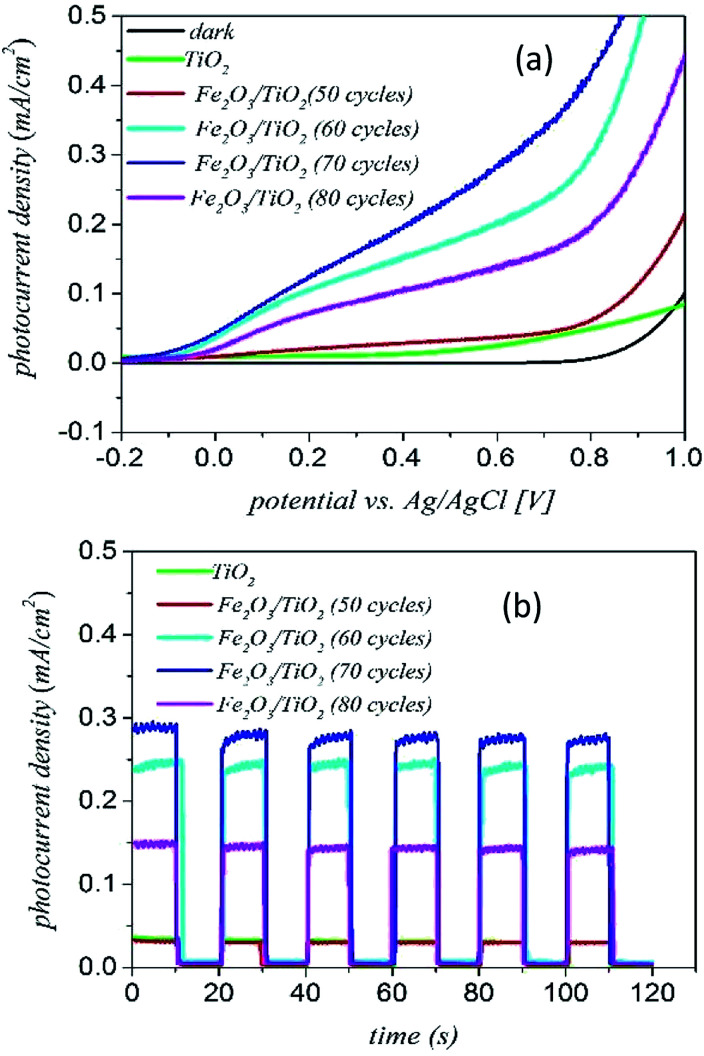
(a) The *J*–*V* measurement curves and (b) chronoamperometry measurements of the α-Fe_2_O_3_ loaded TiO_2_/FTO samples with different deposition cycles.

Fig. 9 shows the results of PEC measurements for the α-Fe_2_O_3_/TiO_2_ samples. The photoelectrochemical properties were carried out using a three-electrode cell with the sample as the working electrode, a Pt wire as the counter electrode and the Ag/AgCl electrode saturated with 3 M KCl as the reference electrode. The electrolyte was 1 M NaOH solution.

Fig. 9(a) shows the photocurrent–voltage (*J*–*V*) curves of various samples for both dark and light illuminations. The dark current curves of all samples are close to zero with no photoelectrochemical activity under the dark condition. The bare sample of TiO_2_ has a small photocurrent density of around 0.04 mA cm^−2^. The *J*–*V* curves of the samples confirm that the current density of the 70 cycles deposition of α-Fe_2_O_3_ gives the highest photocurrent (around 0.3 mA cm^−2^). With increasing the number of deposition cycles to 80 cycles, the current density of the sample decreases. In fact, the 70-cycle deposition of α-Fe_2_O_3_ is the optimum thickness for getting the best water-splitting properties from the composite sample.

Fig. 9(b) shows the transient photocurrent curves of various samples for the ON–OFF period of 20 s. We see that the photocurrent rapidly increases when the light is turned on and it immediately decreased to zero when the light is turned off. According to the results of Fig. 9(b), after about 80 seconds, the photocurrent becomes stable and repeatable. In addition, there is a small slope for the current curve under the light ON condition indicating that the electron–hole recombination is small in the complex of α-Fe_2_O_3_/TiO_2_/FTO.

The main reason for increment of the photocurrent density of the α-Fe_2_O_3_/TiO_2_/FTO electrode relative to the bare TiO_2_ goes back to the higher absorption of the composite material. As shown in Fig. 7, TiO_2_ mainly absorb short wavelength photons below 400 nm. However, adding α-Fe_2_O_3_ to the electrode can extend the optical absorption to the visible spectrum.^46^ As a result, the low energy photons can be absorbed by the α-Fe_2_O_3_ part of the photoelectrode and more photoelectrons can be produced by the composite electrode.^47^

**Fig. 10:**
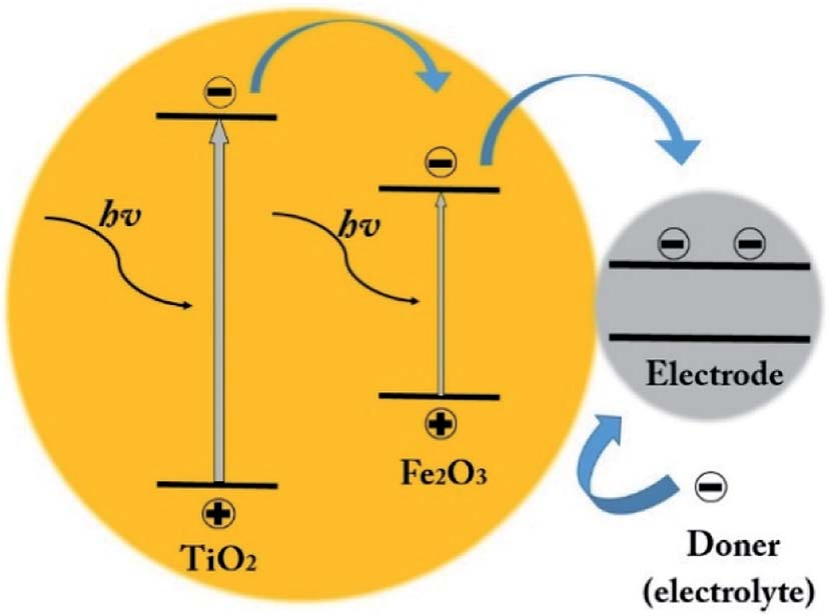
Schematic of the electron–hole generation and the transferring of the photogenerated charges into the photoanode under the visible light illumination.

The next reason is the location of the α-Fe_2_O_3_ edge conduction band compare to TiO_2_ (as shown in Fig. 10). Under UV-vis illumination, electrons can be excited from the valence band (VB) to the conduction band (CB) of the anatase, creating a charge vacancy in the VB. In the absence of the α-Fe_2_O_3_, most of electron–holes are combined quickly.^39,47^

Increasing the coating cycles of hematite (from 50 to 70 cycles), had a significant improvement in current density. According to the results of Fig. 9, the sample of 70-cycle represents the best photocurrent. However, with increasing the number of cycles to 80 cycles, the photocurrent density decreases. Therefore, it is indicated that there was an optimal amount of deposition layer. By increasing the amount of α-Fe_2_O_3_ nanoparticles on the surface of the TiO_2_ nanoparticles, the surface was blocked and light access was greatly reduced, and the production of the electron–hole decreased, so the photocurrent was reduced. So when the amount of α-Fe_2_O_3_ was increased from an optimal amount, because photoelectrons accumulated on α-Fe_2_O_3_, the recombination probability of the electron–holes increased.^48^ So α-Fe_2_O_3_ being as an electron–hole recombination center, and resulted a reduction in optical activity.^49^

**Fig. 11:**
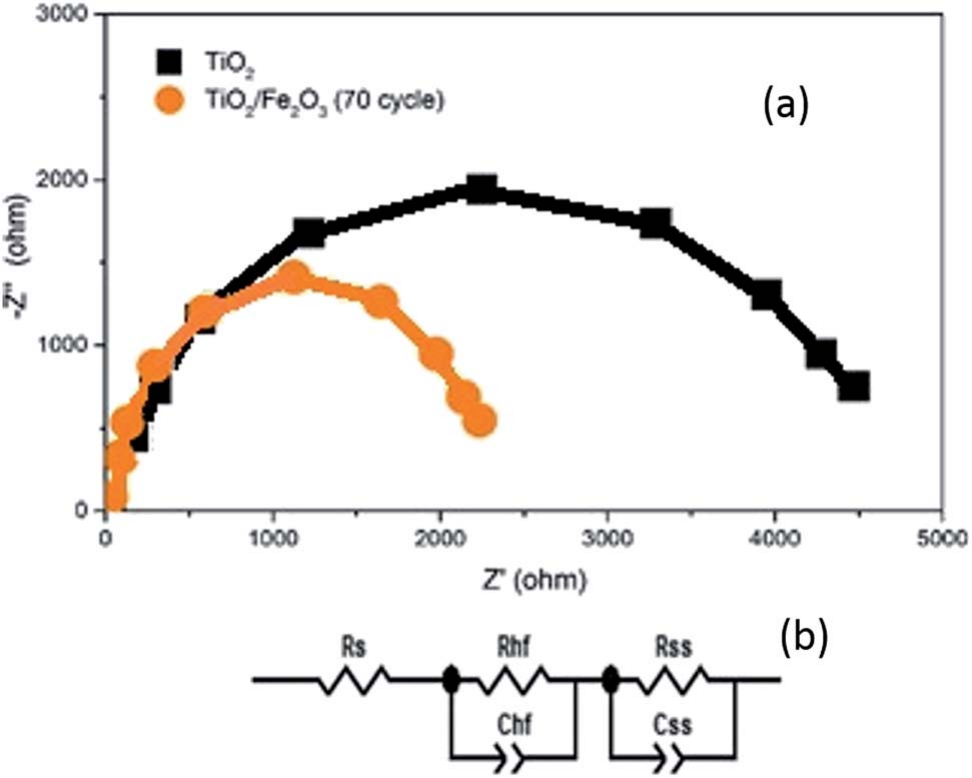
(a) Nyquist plots of TiO_2_/FTO and the sample of α-Fe_2_O_3_/TiO_2_/FTO with 70 cycles. (b) The equivalent circuit used for the fitting of EIS results.

In order to investigate the charge transport properties of the bare TiO_2_ and α-Fe_2_O_3_/TiO_2_ samples, the electrical impedance spectroscopy measurements (EIS) were performed for various samples. The applied frequency range was from 100 mHz to 100 kHz (in a 0.1 M Na_2_SO_4_ electrolyte at the potential of *V* = 0.7 V *versus* the Ag/AgCl reference). The Nyquist plots obtained by the EIS measurements in addition to the equivalent RC circuit for fitting the EIS data are shown in Fig. 11. In the equivalent circuit, *R*_s_ indicates a serial resistance between the FTO substrate and the TiO_2_ layer. *R*_hf_ demonstrates the high frequency resistance due to accumulation and trapping of fast charges throughout the α-Fe_2_O_3_/TiO_2_ composite. *R*_ss_ represents the resistance of charge transfer between the photoanode and the electrolyte. The capacitances *C*_hf_ and *C*_ss_ are relating to the space charge capacitance of the sample and the capacitance associated with the surface states of the photoanode, respectively.

As shown in Fig. 11, the curve of α-Fe_2_O_3_/TiO_2_ photoanode has a smaller semicircle radius than the bare TiO_2_, which indicates that both *R*_hf_ and *R*_ss_ are lower for the α-Fe_2_O_3_/TiO_2_ relative to the bare TiO_2_. This result completely confirms the improvement of charge transfer kinetics of the bare TiO_2_ sample after the Fe_2_O_3_ modification. The faster charge transfer of the 70-cycle sample leads to less surface recombination and improving the photocatalytic activity of this sample relative to all other ones.

It should be mentioned that TiO_2_ has better charge transport properties relative to Fe_2_O_3_, considering the mobility of electrons and the holes in both semiconductors. Therefore, deposition of a thin layer of TiO_2_ under Fe_2_O_3_ in the samples causes that the electrons are better transmitted to the external circuit relative to the case of blank Fe_2_O_3_ on FTO. In fact, the recombination between electron and holes at the interface of TiO_2_/FTO is less than that of Fe_2_O_3_/FTO. Consequently, a higher photocurrent is expected from the stack of Fe_2_O_3_/TiO_2_/FTO relative to that of Fe_2_O_3_/FTO. This issue has clearly been confirmed by other researchers.^42^

## Conclusions

In this work, we reported on the effect of deposition of a thin layer of α-Fe_2_O_3_ on TiO_2_ nanoparticles by the SILAR method for photoelectrochemical water splitting. The formation of α-Fe_2_O_3_ was confirmed by various characterizations including XRD, EDX and FE-SEM spectroscopy. The UV-visible absorption spectrum showed a remarkable enhancement in the absorption of α-Fe_2_O_3_/TiO_2_ relative to the bare TiO_2_. The photoelectrochemical characterizations indicate that all samples of α-Fe_2_O_3_/TiO_2_ have an improved photocurrent compared to the bare TiO_2_. This result is mainly due to the proper bandgap of α-Fe_2_O_3_, which extended the absorption spectrum of the sample into the visible light. Also, the recombination of charge carriers greatly decreased by using α-Fe_2_O_3_ on TiO_2_. We found that there is an optimum number of cycles for the SILAR deposition of α-Fe_2_O_3_, on TiO_2_, which is the 70-cycle exhibiting much better photoelectrochemical activity from α-Fe_2_O_3_/TiO_2_ relative to the bare TiO_2_, with about 7.5 times photocurrent improvement.

## Conflicts of interest

There are no conflicts to declare.

## Supplementary Material
